# Endogenous Production of n-3 Polyunsaturated Fatty Acids Promotes Fracture Healing in Mice

**DOI:** 10.1155/2017/3571267

**Published:** 2017-06-14

**Authors:** Yuhui Chen, He Cao, Dawei Sun, Changxin Lin, Liang Wang, Minjun Huang, Huaji Jiang, Zhongmin Zhang, Dadi Jin, Baiyu Zhang, Xiaochun Bai

**Affiliations:** ^1^Department of Orthopedic, The Third Affiliated Hospital of Southern Medical University, Guangzhou 510665, China; ^2^Department of Orthopedics & Microsurgery, Guangdong Second Provincial General Hospital, Guangzhou 510317, China; ^3^Department of Rehabilitation Medicine, Sun Yat-Sen Memorial Hospital, Sun Yat-Sen University, Guangzhou 510120, China

## Abstract

Bone fracture is a global healthcare issue for high rates of delayed healing and nonunions. Although n-3 polyunsaturated fatty acid (PUFA) is considered as a beneficial factor for bone metabolism, only few studies till date focused on the effects of n-3 PUFAs on fracture healing. In this study, we investigated the effect of endogenous n-3 PUFAs on fracture healing by measuring femur fracture repair in both *fat-1* transgenic mice and WT mice. Proximal femoral fracture model was established in *fat-1* transgenic mice and WT mice, respectively, and then the fracture was analyzed by using X-ray, micro-computed tomography (micro-CT), and histological assessment at 7, 14, 21, 28, and 35 days after fixation. The results showed that compared with WT mice, *fat-1* mice exhibited acceleration in fracture healing through radiographic and histological analysis (18–21 days versus 21–28 days postfracture). Meanwhile, X-ray and micro-CT analysis that showed better remodeling callus formation were in the *fat-1* group compared to WT group. Furthermore, histological analysis revealed that endogenous n-3 PUFAs promoted local endochondral ossification and accelerated the remodeling of calcified calluses after fracture. In conclusion, the present study indicated that endogenously produced n-3 PUFAs promote fracture healing process and accelerate bone remodeling in mice, and supplementation of n-3 PUFAs was positively associated with fracture healing.

## 1. Introduction

Fracture is delineated as a major healthcare problem worldwide within the rapid aging population. The rate of fracture union was about 20%, which still remains as a severe clinical issue even with the improvements of internal fixations in orthopedic management over the past decades [1]. Several researchers have concentrated on diet management for fracture healing, which includes intake of vitamin D and calcium for their modulatory roles in skeletal metabolism [2]. Although vitamin D and calcium deficiency results in high risk of fractures and delay in skeletal development, intake of either vitamin D or calcium accelerates fracture healing due to limited absorption into the digestive tract and in turn balancing the whole body metabolism. Recently, the modulation of fatty acids (FAs) in bone-remodeling process has been reported [3]. For instance, arachidonic acid (AA), a polyunsaturated omega-6 fatty acid, acts as a precursor for the synthesis of lipid signaling molecules such as prostaglandins (PGs) and leukotrienes (LTs). This plays a negative role in fracture healing and AA inhibition, which resulted in the enhancement of fracture healing [4, 5]. According to a clinical study, total fat and animal fat rich in saturated fatty acids might increase the risk of hip fractures in elders [6]. Nevertheless, other FAs like eicosapentaenoic acid (EPA) (20:5; n-3) and docosahexaenoic acid (DHA) (22:6; n-3) were reported to reduce the risk of fractures [7].

Recently, remarkable benefits of n-3 PUFAs on bone metabolism have been reported widely. Bonnet and Ferrari have demonstrated that long-term supplementation of n-3 PUFAs promoted the strength of diaphyseal bone in mice [8]. Consistent with the past investigation [3], our recent study also showed that endogenously produced n-3 PUFAs attenuated the bone loss of ovariectomized mice through bone marrow adipogenesis inhibition [9]. In addition, serum levels of n-3 PUFAs were positively correlated with the bone mineralization in both mice and human, and the fracture risk was increased by intake of saturated FAs while reduced by intake of n-3 PUFAs [3, 6, 10–12].

Although potential benefits of n-3 PUFAs on bone metabolism are promising, no study has yet evaluated the role of n-3 PUFAs on fracture healing or management of diet postfracture. Moreover, *fat-1* transgenic mice can convert n-6 to n-3 PUFAs endogenously; this transgenic mice provide an opportunity to distinguish the regulatory role of n-3 PUFAs [13]. Therefore, the purpose of this present study is to investigate the efficacy of endogenously produced n-3 PUFAs on fracture healing in mice femoral fracture model.

## 2. Materials and Methods

### 2.1. Animals and Diet

All animal experiments were carried out with the approval of Southern Medical University Animal Care and Use Committee in accordance with the ethical treatment of animal guidelines. All surgeries were performed under chloral hydrate and xylazine anesthesia, and all efforts were made to minimize animal suffering. Briefly, *fat-1* transgenic mice were mated with C57BL/6 wild-type mice to obtain *fat-1* positive C57BL/6 mice (*fat-1*) and *fat-1* negative C57BL/6 mice (WT). The genotypes of mice were identified by the presence of *fat-1* gene. Mice were housed under specific pathogen-free (SPF) conditions with day and night cycles of 12 h each. The temperature and humidity was maintained at 25 ± 3°C and 65 ± 5%, respectively, and fed diets containing 10% corn oil. After 12 weeks postnatal, sixty mice were selected and were randomly divided into the following two groups: WT (*n* = 30) and *fat-1* (*n* = 30), 6 mice were randomly selected and harvested at each time point.

### 2.2. Surgical Procedure

Mice were anesthetized by intraperitoneal injection of xylazine (25 mg/kg body weight) and ketamine (75 mg/kg body weight); then a 4 mm medial incision was performed on the right knee, and the patella was dislocated under sterile condition. After a hole (0.45 mm in diameter) was drilled into the intracondylar notch by using a 26-gauge needle, a guide wire (0.2 mm in diameter) was inserted into the femoral intramedullary canal through the needle. The needle was removed, and the guide wire was retained to stabilize the impending fracture. Then, a standardized proximal fracture was manifested on right femur by using a 3-point bending devices. Subsequently, for establishing a simulation model of internal fixation in clinic, a nail (0.45 mm in diameter) was implanted through the guide wire. Finally, the patella was repositioned, and the incision was closed in the 2 layers; the guide wire was removed and extended beyond the needle cut.

### 2.3. Sample Harvest

Mice were anesthetized via intraperitoneal injection with pentobarbital sodium phosphate-buffered saline (PBS) solution of 30 mg/kg body weight and were sacrificed for harvesting right femur samples at weeks 1, 2, 3, 4, and 5 after fracture establishment. The right femurs were then stored at 4°C for further analysis.

### 2.4. X-Ray Radiography

Radiographs were measured by digital radiographic system (Kodak, DirectView DR 3500, Rochester, NY). The right femurs of each group were analyzed at weeks 1, 2, 3, 4, and 5 postfracture, respectively. Fracture healing was evaluated with callus maturity by using Goldberg classification (stage 1: nonunion, stage 2: possible union, and stage 3: complete union) [14]. Mean radiological score of 5 weeks was calculated for both groups.

### 2.5. Micro-Computed Tomography (Micro-CT)

Micro-CT scan was used for measuring with ZKKS-MCT-III micro-CT system (Guangzhou Zhongke Kaisheng Medical Technology Company Ltd., Guangzhou, China). Each sample was placed and then secured with foam board to avoid shifting during scan.

### 2.6. Histological Analysis

At 1, 2, 3, 4, and 5 weeks postfracture, right femurs were collected and were decalcified by 19% EDTA solution for about 2–4 weeks and then were dehydrated through successive grades of ethanol, rinsed with xylene, and embedded in paraffin. Sections of 4 *μ*m were prepared sagittally at the fracture site. The sections were stained by Safranin O/Fast Green staining (SO/FG) and were observed under a light microscope (Olympus BX51, Japan).

### 2.7. Statistical Analysis

Data were expressed as mean ± SD, and Student's *t*-test was performed to analyze statistical significance. Representative experiments were presented in the Results section and figures of this study. Statistical significance was achieved when *p* < 0.05.

## 3. Results

### 3.1. X-Ray Analysis

**Figure 1:**
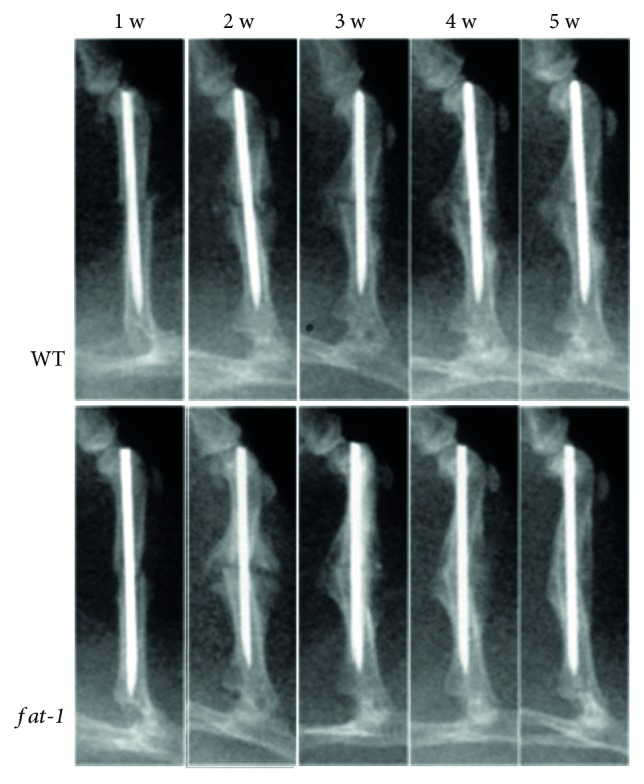
X-ray images of WT and *fat-1* mice at 1, 2, 3, 4, and 5 weeks postfracture.

The results revealed that in the same diet conditions, no significant difference was presented in the body weight of mice in the two groups (*p* > 0.05, data not shown). The fracture healing of each group was assessed by X-ray analysis at 1, 2, 3, 4, and 5 weeks postsurgery (Figure 1). X-ray radiographs (Figure 1) demonstrated that the fracture line was observed in the WT group at 4 weeks postfracture, but was barely detectable in *fat-1* group at the same time. In addition, results showed that callus formation in *fat-1* group reached peak at 2 weeks postfracture, which was earlier than that in WT group (3 weeks postfracture). Calcified calluses and bone unions were observed in WT mice at 3 and 4 weeks postfracture, respectively, but was accelerated in *fat-1* mice (2 and 3 weeks postfracture). Moreover, remodeling of calcified callus was initiated at 3 weeks postfracture in *fat-1* group when compared to WT group (5 weeks postfracture).

**Figure 2:**
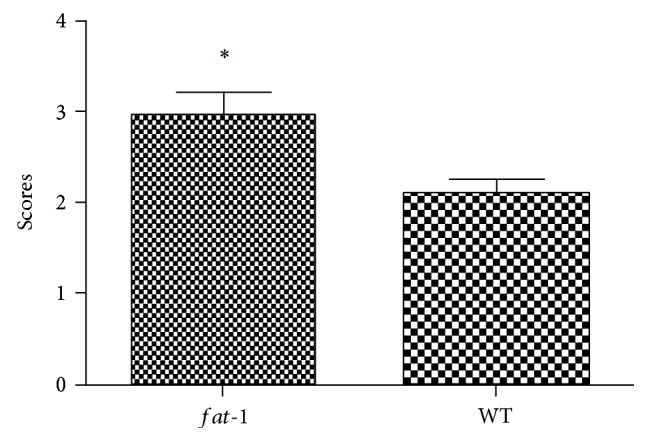
The Goldberg scores of two groups at 5 weeks postfracture. ^∗^  *p* < 0.05.

Simultaneously, for a better measurement of fracture healing, Goldberg classification was used to reveal the callus maturity in the two groups at 5 weeks postfracture [14]. Results showed that the score of *fat-1* group (*n* = 6 for each time point) was significantly higher than that of WT group (Figure 2). Taken together, these data suggested remarkable acceleration of fracture healing in *fat-1* mice and the endogenously produced n-3 PUFAs promoted the fracture healing process of mice.

### 3.2. Micro-CT Measurement

**Figure 3:**
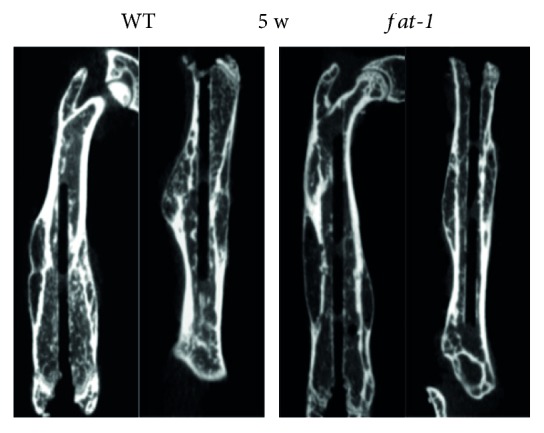
Bone remodeling of WT and *fat-1* mice at 5 weeks postfracture.

To measure the mineralization of fracture 3D reconstruction of the fractured femur was performed at 5 weeks postfracture, respectively. Though all the fracture healing process has been completed at this time point, the cortical remodeling and cortical bone mass in the fracture area of *fat-1* group presented a significant enhancement compared to those of WT group (Figure 3). These results suggested that in addition to the acceleration of bony regeneration, the endogenously produced n-3 PUFAs enhanced the strength of new bone as well.

### 3.3. Histological Analysis

**Figure 4:**
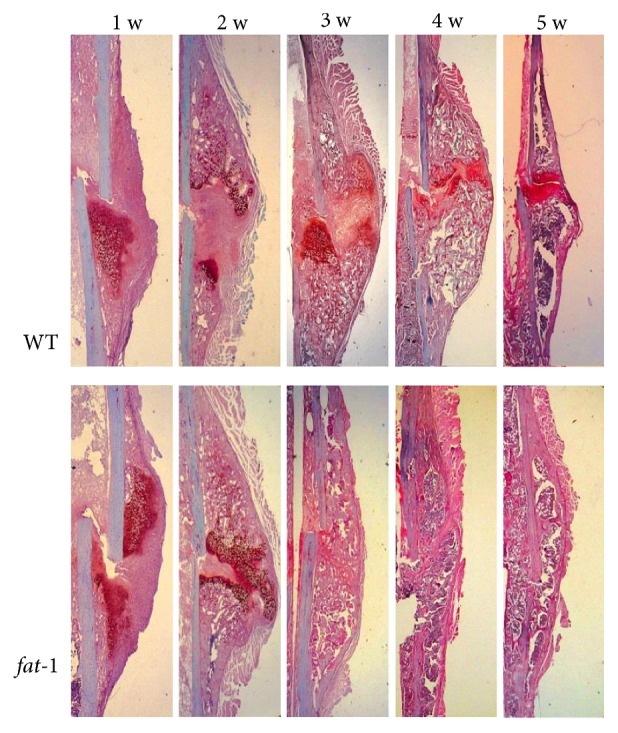
Histological measurement of WT and *fat-1* mice at 1, 2, 3, 4, and 5 weeks postfracture.

To evaluate the formal healing process postfracture of each group, SO/FG stain of each group was performed at 1, 2, 3, 4, and 5 weeks after fracture (Figure 4). The fracture healing process of mice undergoing callus formation, endochondral ossification, and remodeling was described previously [15]. In our observations, mature woven bone and continuous callus formation were presented in the fracture area of *fat-1* group at about 3 weeks postfracture, but early formation of these in WT mice (5 weeks) suggested a promotion of endochondral ossification during the fracture healing process of *fat-1* mice. In addition, clear remodeling was observed in fracture area of *fat-1* mice at 3 weeks postfracture, while remodeling was initiated in WT mice at 4 weeks postfracture. Therefore, our results showed that *fat-1* mice exhibited acceleration in callus formation, endochondral ossification, and remodeling process compared to WT group.

## 4. Discussion

Recently, the positive roles of n-3 PUFAs in bone remodeling and antiosteoporosis have been demonstrated [3, 16]. Simultaneously, the reduction of n-6/n-3 PUFA ratio in the serum level was positively correlated with mammalian bone mineral density (BMD) and mechanical loading as well [17]. Although former studies have already provided new insights into the effects of n-3 PUFAs on bone metabolism, only few studies focused on the effects of n-3 PUFAs on fracture repair. Therefore, in this present study, we utilized the transgenic *fat-1* mice to determine if the endogenously produced n-3 PUFAs could accelerate fracture healing. To our knowledge, this study is the first study to reveal the effect of endogenous n-3 PUFAs on fracture healing by establishing a transgenic animal model.

Omega-3 polyunsaturated fatty acids are a group of essential fatty acids which cannot be synthesized in sufficient amounts in the body, therefore, they can be supplemented through human diet [18]. The potential health benefits of these have been demonstrated in the past decades, which reduced the risk of coronary heart disease and fracture, and other potential benefits in the prevention and treatment of autoinflammatory disorders, and insulin resistance [19]. The *fat-1* mice are accomplished by transgenic technique and can endogenously converse n-6 PUFAs to n-3 PUFAs, thus were compared to WT mice. The serum ratio of n-6/n-3 PUFAs was significantly lower in *fat-1* transgenic mice compared with control that had sufficient n-6 PUFA intake [13]. In the present study, to eliminate the potential interference factors from diets, *fat-1* mice were chosen to investigate the efficacy of n-3 PUFAs on fracture repair.

Similarly, our results showed a remarkable acceleration of healing time in *fat-1* mice compared to WT mice both radiologically as well as histologically [8]. Meanwhile, the terminal BMD and remodeling in the facture area of *fat-1* mice enhancement suggested that endogenous n-3 PUFAs can promote fracture healing and optimized the final mineralization of fracture in mice.

Although the present study provided advanced evidence to indicate the positive role of n-3 PUFAs in fracture repair and bone metabolism, the clear mechanism of n-3 PUFAs in modulating the bone repair was absent in our results; this point was the major limitation of our investigation. Therefore, in our future works, the potential signaling that is correlated with skeletal and FA metabolism should be clearly distinguished.

Some studies suggested that AA, a classic n-6 PUFA, has been reported to inhibit endochondral ossification and bone mineralization through activating inflammatory factors like PGE-2 and LTs [20, 21]. In addition, diet supplementation of AA promoted bone formation and reduced the postmenopausal bone loss [22]. Moreover, serum phospholipid levels of n-3 PUFAs were positively associated with BMD in healthy populations and high serum levels of n-3 PUFAs reduced the risk of osteoporosis in postmenopausal women [23, 24]. Bone-remodeling process was considered to be the vital factor in the maintenance of adult bone mass [25, 26], and consistent with these investigations, our study results demonstrated acceleration of bone remodeling and callus calcification in *fat-1* mice postfracture.

Nevertheless, numerous evidences have proved the vital role of inflammation in fracture healing, and the knockdown of inflammatory factors in mice resulted in the delay of bone repair [27]. Moreover, the local stromal cell recruitment and angiogenesis in the fracture area suppressed the inflammation and then reduced osteogenesis, which lead to the retardation of fracture healing [28]. Therefore, the positive role of n-3 PUFAs in skeletal metabolism is a complex issue and need to be studied deeply. However, most of the investigators believed in the hypothesis that n-6 PUFAs convert to n-3 PUFAs endogenously or intake of n-3 PUFAs may promote bone repair by inhibiting inflammation. Although the mechanism still needed further research, n-3 PUFAs has been shown to reduce the formation of PGE-2 and promotes bone formation through enhancing the expression of insulin-like growth factors, which are powerful growth stimulators for bone remodeling [29, 30]. Meanwhile, muscle-derived n-3 PUFAs have also been shown to increase the digestive calcium absorption and decrease the release of inflammatory cytokines of osteoclasts [29].

In the present study, endogenous conversion of n-6 to n-3 PUFAs promotes terminal differentiation of endochondral ossification and accelerates remodeling of calcified callus in fracture healing of *fat-1* mice. With multiple beneficial effects of n-3 PUFAs in cardio-protection and insulin resistance reduction [16], intake of these has potential application in fracture healing. Our study provides compelling evidence to support the dietary supplementation of n-3 PUFAs in fractures. Meanwhile, a lower n-6/n-3 PUFA ratio should be considered as an impact factor in the diet management of patient with bone fracture.

## 5. Conclusion

We investigated the effect of endogenous n-3 PUFAs on fracture healing by measuring femur fracture repair in both *fat-1* transgenic mice and WT mice in this study. Our results showed that compared with WT mice, *fat-1* mice exhibited accelerations in healing the fracture. Meanwhile, better endochondral ossification and remodeling callus were presented in *fat-1* mice postfracture. In conclusion, the present study indicated that endogenously produced n-3 PUFAs promoted fracture healing process and accelerated bone remodeling, which suggested that supplementation of n-3 PUFAs may play a positive role in fracture healing.
